# Supplementary material for: Mechanisms governing the pioneering and redistribution capabilities of the non-classical pioneer PU.1
Source: Nat Commun. 2020 Jan 21;11:402. doi: 10.1038/s41467-019-13960-2 (PMC6972792; doi:10.1038/s41467-019-13960-2)
Supplement: Supplementary file 7 — Source data [file 41467_2019_13960_MOESM7_ESM.zip › Source_Data/Figure6/Figure6B_MotifScanOutput/ETS-specific/homerResults/motif35.similar.html]

motif35

## Information for motif35

C
T
A
G
G
T
A
C
C
T
A
G
A
G
T
C
G
C
T
A
C
A
G
T
A
C
T
G
A
G
T
C
A
C
T
G
A
G
T
C
T
G
C
A
C
T
A
G
  
Reverse Opposite:  

G
A
T
C
A
C
G
T
C
T
A
G
T
G
A
C
C
T
A
G
A
G
T
C
G
T
C
A
C
G
A
T
A
C
T
G
A
G
T
C
C
A
T
G
A
G
T
C
  

|  |  |
| --- | --- |
| p-value: | 1e-26 |
| log p-value: | -6.172e+01 |
| Information Content per bp: | 1.728 |
| Number of Target Sequences with motif | 234.0 |
| Percentage of Target Sequences with motif | 3.06% |
| Number of Background Sequences with motif | 497.4 |
| Percentage of Background Sequences with motif | 1.23% |
| Average Position of motif in Targets | 106.0 +/- 55.7bp |
| Average Position of motif in Background | 96.9 +/- 64.7bp |
| Strand Bias (log2 ratio + to - strand density) | 0.2 |
| Multiplicity (# of sites on avg that occur together) | 1.11 |
| Motif File: | file (matrix) reverse opposite |

### Similar de novo motifs found

|  |  |  |  |  |  |  |  |
| --- | --- | --- | --- | --- | --- | --- | --- |
| Rank | Match Score | Redundant Motif | P-value | log P-value | % of Targets | % of Background | Motif file |
| 1 | 0.979 | A T G C A C G T C T A G G T A C C T A G A G T C T G C A A C G T A C T G A G T C A C T G A G T C T G C A | 1e-24 | -55.629041 | 3.04% | 1.30% | motif file (matrix) |
| 2 | 0.983 | C T A G T G A C C T A G A G T C G T C A A C G T A C T G A G T C A C T G A G T C T G C A T A C G A C G T T A C G | 1e-20 | -47.251258 | 3.74% | 1.89% | motif file (matrix) |
| 3 | 0.939 | C T A G T G A C A C T G A T G C G T C A C A G T T A C G A T G C A C T G | 1e-18 | -42.386346 | 3.81% | 2.03% | motif file (matrix) |
| 4 | 0.880 | G T A C A C T G A G T C G T C A C G A T A C T G A G T C A C T G | 1e-14 | -34.295481 | 4.44% | 2.67% | motif file (matrix) |
